# Draft Genome Sequence of an *Enterococcus faecalis* ATCC 19433 Siphovirus Isolated from Raw Domestic Sewage

**DOI:** 10.1128/genomeA.01179-16

**Published:** 2017-01-19

**Authors:** Tasha M. Santiago-Rodriguez, Melissa Ly, David T. Pride, Gary A. Toranzos

**Affiliations:** a Center for Applications in Biotechnology, California Polytechnic State University, San Luis Obispo, California, USA; b Department of Biological Sciences, California Polytechnic State University, San Luis Obispo, California, USA; c Department of Pathology, University of California, La Jolla, California, USA; d Department of Medicine, University of California, La Jolla, California, USA; e Environmental Microbiology Laboratory, Biology Department, University of Puerto Rico, San Juan, Puerto Rico

## Abstract

We previously isolated and characterized an *Enterococcus faecalis* ATCC 19433 siphovirus from raw domestic sewage as a viral indicator of human fecal pollution. Here, we report the draft genome sequence of this bacteriophage.

## GENOME ANNOUNCEMENT

Previous studies have characterized phages specific for *Enterococcus faecalis* ATCC 19433 isolated from raw domestic sewage as microbial indicators of fecal contamination (1). Morphological characterization has shown that *E. faecalis* ATCC 19433 phages possess long noncontractile tails (200 nm) and icosahedral capids (60 nm) typical of siphoviruses (2). Here, we report the draft genome sequence of an *E. faecalis* ATCC 19433 phage isolated from raw domestic sewage.

One milliliter (10^9^ PFU/ml) of the *E. faecalis* ATCC 19433 phage was filtered sequentially using 0.45- and 0.2-μm filters (VWR, Radnor, PA, USA) and purified on a cesium chloride (CsCl) density gradient (3). One milliliter of the CsCl fraction was purified on Amicon YM-100 protein columns (Millipore, Billerica, MA) and treated with DNase I. DNA was isolated using a Qiagen UltraSens virus kit (Qiagen, Valencia, CA, USA), amplified using GenomiPhi HY MDA (GE Healthcare, Pittsburgh, PA, USA), and fragmented to 200 to 400 bp using a Bioruptor (Diagenode, Denville, NJ, USA). Libraries were created using the Ion Plus fragment library kit and sequenced using 314 Chips on an Ion Torrent Personal Genome Machine (Life Technologies, Grand Island, NY, USA), producing an average read length of 206 bp. Reads were trimmed according to modified Phred scores of 0.5 using CLC Genomics Workbench 4.65 (Cambridge, MA, USA). After low-complexity and ambiguous reads were removed and the pool cleared of contaminating cellular DNA (ftp://ftp.ncbi.nlm.nih.gov/genomes/H_sapiens/), the remaining reads were assembled using CLC Genomics Workbench 4.65 based on 98% identity, with a minimum of 50% read overlap (3, 4). Open reading frames (ORFs) were predicted using the RAST annotation server with default parameters, followed by manual curation.

Reads were assembled into a contig of 37,933 bp (1,136× coverage). Sixty-one ORFs but no tRNAs were found, and the ends of the genome were not determined (i.e., the sequence was not shown to be complete by PCR from ends). The initiation codons of 55, three, and two predicted ORFs were AUG, GUG, and UUG, respectively. Fifty-five predicted ORFs had unannotated but valid ribosomal-binding sites (RBS). The genome could be divided into functional clusters, including morphogenesis (capsid, tail, tail tape-measure, and head-tail joining genes), replication (DNA polymerase and primase/helicase genes), and host lysis (lysin and holing genes). Over 40 hypothetical proteins were identified. This phage showed an identity of 94% to* Enterococcus* phage EfaCPT1 (accession no. JX193904) (e-value = 0.0), with a major unique region between bp 11576 and 15516. BLASTn results of the bp 11576 to 13096 region showed an identity of 45 to 46% (e-value = 0.0) to *Enterococcus* phage Ec-ZZ2 (accession no. AKG94426) and IME-EF4 (accession no. YP_009004356) minor tail proteins. The region from 13097 to 15516 bp showed an identity of 53% to *Enterococcus* phage IME_EF3 putative minor tail protein (accession no. YP_009008950) (e-value = 8.0e^-95^). The results suggest that these regions may be essential for host recognition and may be unique for *E. faecalis* ATCC 19433 phages.

### Accession number(s).

The complete draft genome sequence of this *E. faecalis* ATCC 19433 phage is available in GenBank under the accession number KX284704.
